# Priming for enhanced *ARGONAUTE2* activation accompanies induced resistance to cucumber mosaic virus in *Arabidopsis thaliana*

**DOI:** 10.1111/mpp.13005

**Published:** 2020-10-19

**Authors:** Sugihiro Ando, Michal Jaskiewicz, Sei Mochizuki, Saeko Koseki, Shuhei Miyashita, Hideki Takahashi, Uwe Conrath

**Affiliations:** ^1^ Graduate School of Agricultural Science Tohoku University Sendai Japan; ^2^ Department of Plant Physiology Aachen Biology and Biotechnology RWTH Aachen University Aachen Germany

**Keywords:** chromatin modification, cucumber mosaic virus, defence priming, histone modification, induced resistance, RNA silencing, systemic acquired resistance

## Abstract

Systemic acquired resistance (SAR) is a broad‐spectrum disease resistance response that can be induced upon infection from pathogens or by chemical treatment, such as with benzo‐(1,2,3)‐thiadiazole‐7‐carbothioic acid *S*‐methyl ester (BTH). SAR involves priming for more robust activation of defence genes upon pathogen attack. Whether priming for SAR would involve components of RNA silencing remained unknown. Here, we show that upon leaf infiltration of water, BTH‐primed *Arabidopsis thaliana* plants accumulate higher amounts of mRNA of *ARGONAUTE (AGO)2* and *AGO3*, key components of RNA silencing. The enhanced *AGO2* expression is associated with prior‐to‐activation trimethylation of lysine 4 in histone H3 and acetylation of histone H3 in the *AGO2* promoter and with induced resistance to the yellow strain of cucumber mosaic virus (CMV[Y]). The results suggest that priming *A. thaliana* for enhanced defence involves modification of histones in the *AGO2* promoter that condition *AGO2* for enhanced activation, associated with resistance to CMV(Y). Consistently, the fold‐reduction in CMV(Y) coat protein accumulation by BTH pretreatment was lower in *ago2* than in wild type, pointing to reduced capacity of *ago2* to activate BTH‐induced CMV(Y) resistance. A role of *AGO2* in pathogen‐induced SAR is suggested by the enhanced activation of *AGO2* after infiltrating systemic leaves of plants expressing a localized hypersensitive response upon CMV(Y) infection. In addition, local inoculation of SAR‐inducing *Pseudomonas syringae* pv. *maculicola* causes systemic priming for enhanced *AGO2* expression. Together our results indicate that defence priming targets the *AGO2* component of RNA silencing whose enhanced expression is likely to contribute to SAR.

## Graphical abstract

In *Arabidopsis thaliana*, activation of SAR to CMV includes modification to histones in the *AGO2* promoter, which seems to condition *AGO2* for enhanced expression upon challenge.

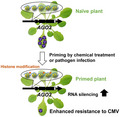

## 1 INTRODUCTION

During evolution, plants developed a multiplicity of interacting and partly overlapping defence responses to fight microbial infection. One set of plant defence responses is induced upon recognition of microbe‐associated molecular patterns (MAMPs), which eventually may cause MAMP‐triggered immunity (MTI). Although MTI often wards off multiple pathogens, the defence response can be suppressed, and possibly overcome, by pathogens that synthesize and secrete adequate defence‐suppressing effector molecules. To avoid infection by MTI‐suppressing pathogens, plants developed an additional defence strategy when they evolved so‐called resistance (*R*) genes. The encoded R proteins, directly or indirectly via guard proteins, can detect effector presence and when they do so they initiate defence (Dodds and Rathjen, 2010). Effector‐triggered immunity (ETI) is often associated with very robust defence reactions such as the hypersensitive response (Jones and Dangl, 2006).

Following activation of MTI and/or ETI, plants frequently build up an enhanced capacity to activate defence responses not only in the area of initial attack but also in the distal, uninoculated parts of the plant. The enhanced defence capacity, which was referred to as defence priming (Conrath et al., 2002, 2006, 2015; Spoel and Dong, 2012), accompanies various types of induced disease resistance (Conrath et al., 2002, 2006, 2015). They include rhizobacteria‐induced systemic resistance (Pieterse et al., 2014), β‐aminobutyric acid‐induced resistance (Zimmerli et al., 2000), and systemic acquired resistance (SAR). The latter is activated upon infection from necrotizing pathogens (Fu and Dong, 2013) or after treatment with various chemicals (Beckers and Conrath, 2007), and wards off a broad spectrum of biotrophic pathogens (Glazebrook, 2005). In addition to serving as a paradigm for studying signal transduction, SAR has practical value as well (Ryals et al., 1996; Conrath et al., 2002).

In *Arabidopsis thaliana*, priming is associated with an elevated level of MAMP‐recognition receptors (Tateda et al., 2014), accumulation of dormant signalling enzymes, such as mitogen‐activated protein kinases 3 and 6 (Beckers et al., 2009), transcription factor HsfB1 activity (Pick et al., 2012), and alterations to chromatin in the promoters of defence genes, such as *WRKY6*, *WRKY29*, *WRKY53*, and *PR1* (Jaskiewicz et al., 2011; Baum et al., 2019). The priming‐associated modification of chromatin in the 5′‐regulatory (promoter) region of those defence‐related genes involves trimethylation of lysine residue 4 in histone H3 (H3K4me3), acetylation of lysine 9 in the same histone (H3K9ac), and acetylation of lysine 5, 8, and 12 in histone H4 (H4K5ac, H4K8ac, and H4K12ac) (Jaskiewicz et al., 2011). Recently, it has been shown that the priming‐linked modification of histones and DNA in defence gene promoters is associated with the formation of nucleosome‐depleted DNA sites that can be identified by formaldehyde‐assisted isolation of regulatory DNA elements (Baum et al., 2019, 2020). Other molecular mechanisms of defence priming remain unknown.

Many chemical signals are associated with the induction of SAR (Dempsey and Klessig, 2012). While the role of some of these signals in SAR has been under much debate (Dempsey and Klessig, 2012), it is well appreciated that salicylic acid (SA) and pipecolic acid are required for defence priming and SAR in *A. thaliana* and some other plant species (Gaffney et al., 1993; Bernsdorff et al., 2016; Hartmann and Zeier, 2019). Consistently, both phenomena can be induced by treatment with SA, pipecolic acid and also various other chemical compounds (Ward et al., 1991; Beckers and Conrath, 2007; Návarová et al., 2012). Amongst them, the synthetic SA analogue benzo‐(1,2,3)‐thiadiazole‐7‐carbothioic acid *S*‐methyl ester (BTH; acibenzolar‐*S*‐methyl) was reported to induce defence priming and SAR (Katz et al., 1998) and to provide protection from various crop diseases in the field (Görlach et al., 1996; Ryals et al., 1996; Beckers and Conrath, 2007). Therefore, the compound was promising for practical agronomic use, and, in 1996, BTH was introduced as a plant activator (Ruess et al., 1996) with trade names such as Bion and Actigard.

RNA silencing has evolved as an antiviral defence strategy in plants (Lindbo et al., 1993; Baulcombe, 2004; Wang et al., 2012; Pumplin and Voinnet, 2013; Ando et al., 2019). During replication of a viral RNA genome, double‐stranded RNA (dsRNA) is produced and subsequently processed by dicer‐like (DCL) enzymes, resulting in the generation of virus‐specific small‐interfering RNAs (siRNAs) (Diaz‐Pendon et al., 2007; Seo et al., 2013). These siRNAs are loaded on Argonaute (AGO) proteins that, together with additional proteins, form a multiprotein complex called the RNA‐induced silencing complex (RISC). RISC degrades viral RNAs in a sequence‐specific manner (Pantaleo et al., 2007). To amplify the RNA silencing signal, host RNA‐dependent RNA polymerase (RDR) produces dsRNA using the truncated viral RNA as a template. The resulting dsRNAs are digested by DCL protein to form secondary siRNA molecules for further digestion of viral RNAs by RISC (Incarbone and Dunoyer, 2013).

In the RNA silencing mechanism, AGO1 and AGO2 seem to have nonredundant and mutually supporting functions in the defence to viruses (Harvey et al., 2011; Alvarado and Scholthof, 2012; Seo et al., 2013). AGO2 has an additional role in the immune response to *Pseudomonas syringae* pv. *tomato* in that it recruits the complementary strand microRNA miR393b* to modulate the exocytosis of antimicrobial pathogenesis‐related proteins (Zhang et al., 2011). However, whether AGO proteins or other components of RNA silencing have a role in defence priming and SAR remained unknown. Here, we merged our groups’ expertise in defence priming and virology/RNA silencing and we show that AGO2 has a role in BTH‐induced defence priming and SAR.

## 2 RESULTS

### 2.1 BTH pretreatment enhances the activation of *AGO2* and *AGO3* expression

**FIGURE 1:**
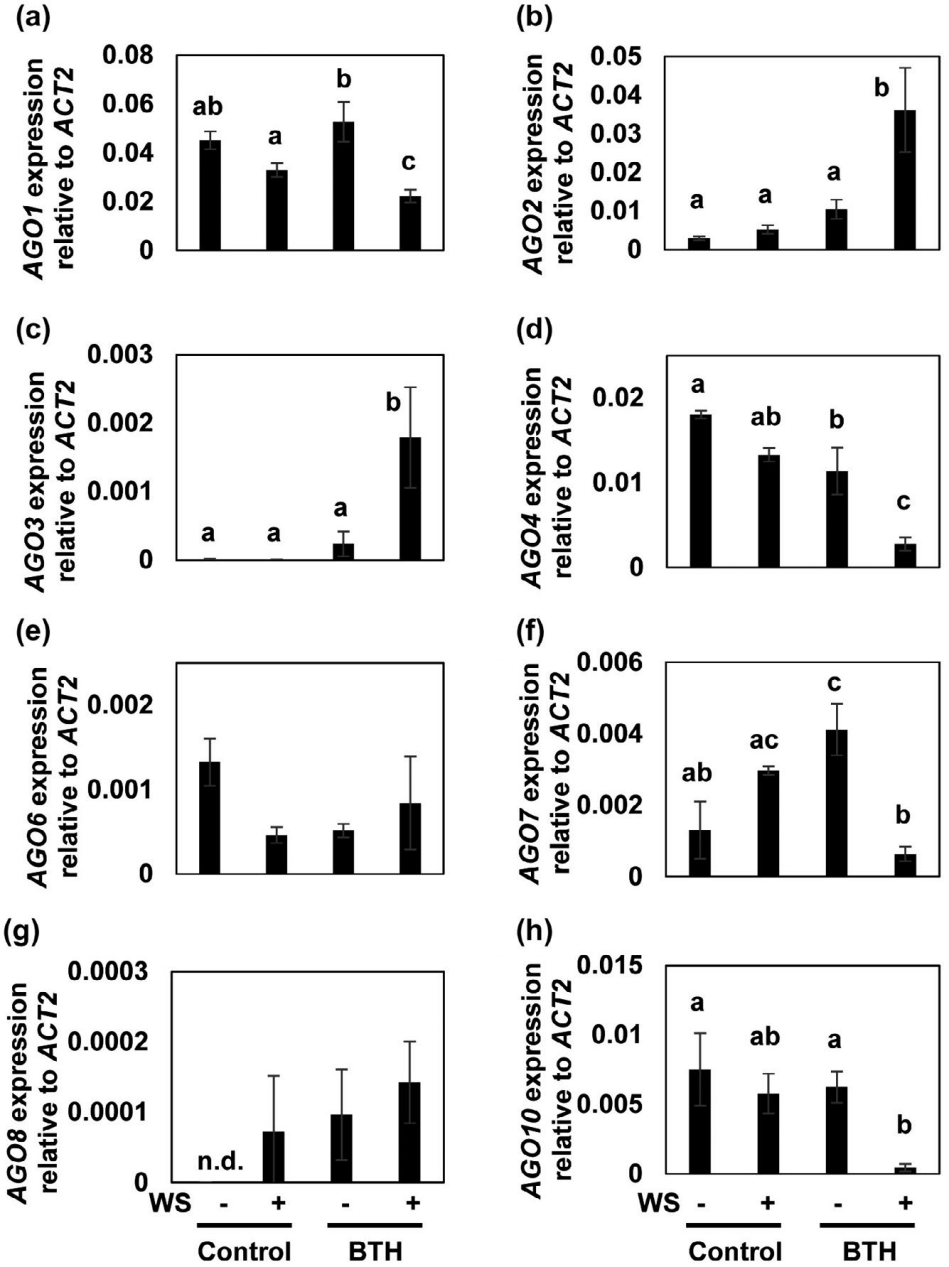
Influence of benzo‐(1,2,3)‐thiadiazole‐7‐carbothioic acid *S*‐methyl ester (BTH) treatment on *AGO* expression. Leaves of 6‐week‐old *Arabidopsis thaliana* plants were sprayed with a solution of wettable powder (WP) without (control) or with BTH (100 µM). After 3 days, leaves of half of the plants were infiltrated with water (water stress, +WS) or left without infiltration (−WS). Three hours later, leaves were harvested, and RNA extracted and subjected to analysis of mRNA transcript abundance of the indicated *AGO* genes. Data were normalized to *ACTIN2* mRNA transcript abundance. Experiments were performed at least three times. A representative result is shown. Different letters denote significant differences between treatments (Tukey–Kramer test, *n* = 3, *p* < .05). *ACT2*, *ACTIN2*; n.d., not detected

**FIGURE 2:**
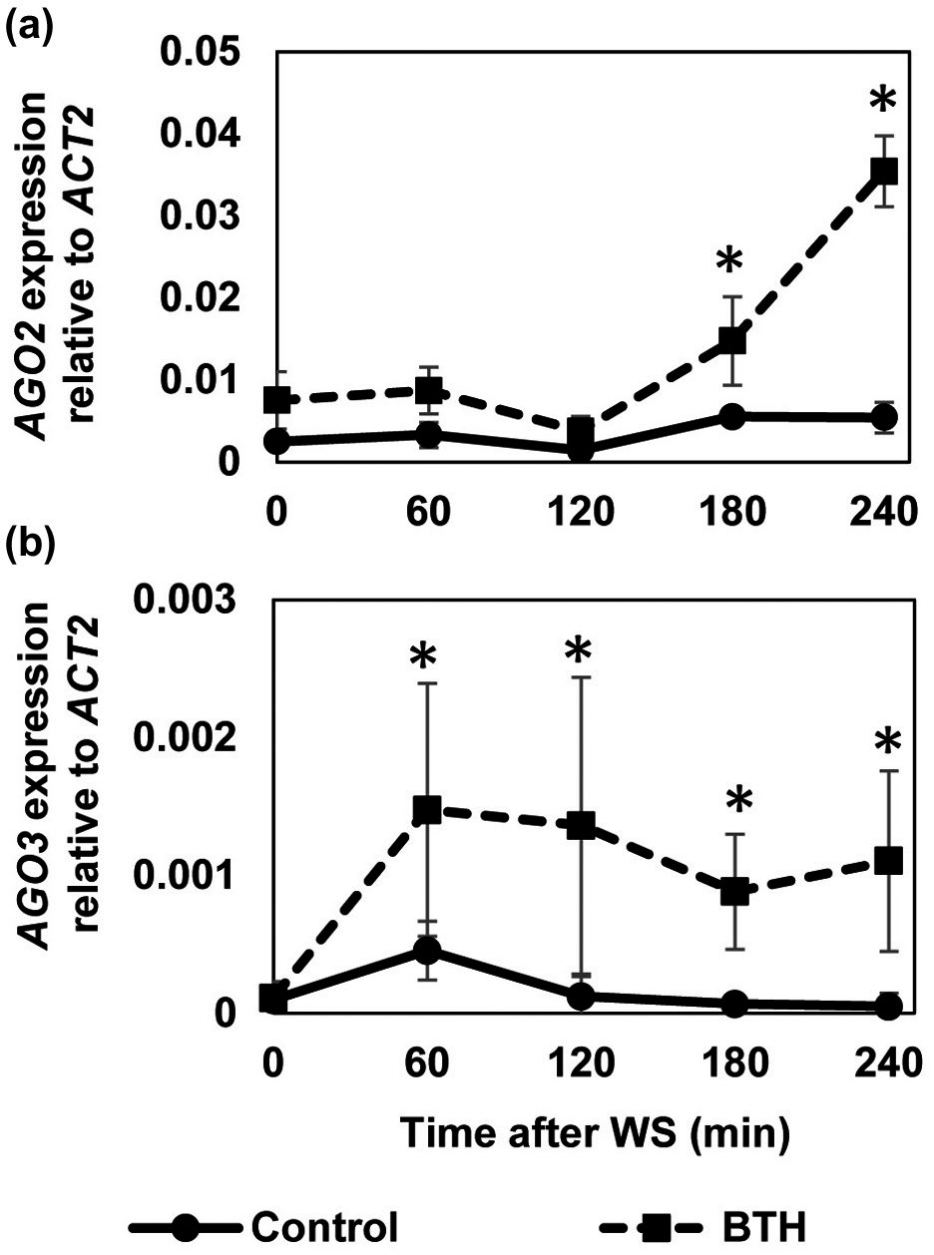
Time course of *AGO2* and *AGO3* expression in benzo‐(1,2,3)‐thiadiazole‐7‐carbothioic acid *S*‐methyl ester (BTH)‐pretreated and control leaves after leaf infiltration. Plants were treated and analysed as in Figure 1. Asterisks denote significant differences among treatments (Student's *t* test, *n* = 3 (a) or 6 (b), *p* < .05). *ACT2*, *ACTIN2*. WS, leaf infiltration with water

In the *A. thaliana* genome, there are four *DCL* genes (*DCL1–4*), six *RDR* genes (*RDR1–6*), and 10 *AGO* genes (*AGO1–10*). To investigate whether there is a link between RNA silencing and defence priming during SAR we treated *A. thaliana* plants with BTH and investigated whether genes in the RNA silencing machinery are directly activated or primed for enhanced activation. To check for the presence of defence priming, we sprayed plants with a wettable powder (WP) formulation of BTH. Treatment with WP devoid of BTH served as a control. Three days later, we challenged the plants by leaf‐infiltration with water, which activates defence genes (Figure S1) (Kohler et al., 2002; Beckers et al., 2009; Jaskiewicz et al., 2011). Among the RNA silencing‐associated genes assayed, the infiltration‐induced expression of *AGO2* (At1g31280) and *AGO3* (At1g31290) was enhanced in leaves of BTH‐pretreated plants (Figure 1b,c) at 3 and 1 hr, respectively (Figure 2). Notably, *AGO2* and *AGO3* showed different expression patterns, suggesting different roles of these two genes in BTH‐induced SAR.

In contrast to enhanced *AGO2* and *AGO3* expression, *AGO7*, which belongs to the same AGO clade (Seo et al., 2013), showed a reduced accumulation of mRNA transcript in BTH‐pretreated plants after challenge (Figure 1f). The basal expression of *AGO6* was apparently reduced by the infiltration of leaves, after BTH treatment, and possibly a combination of the two treatments, although the reduction was not significant for the combined treatment (Figure 1e). Expression of *AGO8*, with a role in the direct defence to herbivory (Pradhan et al., 2017), seemed to be similar in all the samples assayed (Figure 1g). Notably, the mRNA transcript abundance of *AGO1*, *AGO4*, and *AGO10* was also markedly reduced by leaf infiltration of BTH‐pretreated plants (Figure 1a,d,h). The expression of *AGO5* and *AGO9* was below the detection limit (data not shown).

When we assayed the accumulation of mRNA transcript of *DCL* genes, we found that the expression of *DCL1* and *DCL4* was similar to that of *AGO7* (Figure S2a,d). BTH treatment did not significantly affect *DCL2* and *DCL3* expression in the uninfiltrated controls (Figure S2b,c). Except for the challenged control sample (control, WS+), *RDR1* expression (Figure S3a) was like *AGO1* (Figure 1a) and *AGO7* (Figure 1f), *DCL1* (Figure S2a), *DCL2* (Figure S2b), and *DCL4* (Figure S2d). Moreover, the expression of *RDR2* and *RDR6* was decreased upon leaf infiltration, and *RDR2* expression lower in BTH‐treated leaves compared to untreated controls (Figure S3b,f). When compared to other *RDR*s the expression of *RDR3*, *RDR4,* and *RDR5* was generally low and did not increase after BTH treatment (Figure S3c–e).

Together we found that, among the RNA‐silencing components assayed, *AGO2* and *AGO3* expression was enhanced in infiltrated leaves of BTH‐primed plants (Figure 1b,c). Because the overall abundance of *AGO2* mRNA was c.20‐times higher than the transcript of *AGO3* (Figure 1b,c), we decided to investigate the role of *AGO2* in the BTH‐primed plant defence response. However, this does not exclude the fact that AGO3 might also be important to BTH‐induced SAR.

### 2.2 Inoculation with CMV(Y) or *P. syringae* pv. *maculicola* induces systemic priming for enhanced activation of *AGO2* expression

**FIGURE 3:**
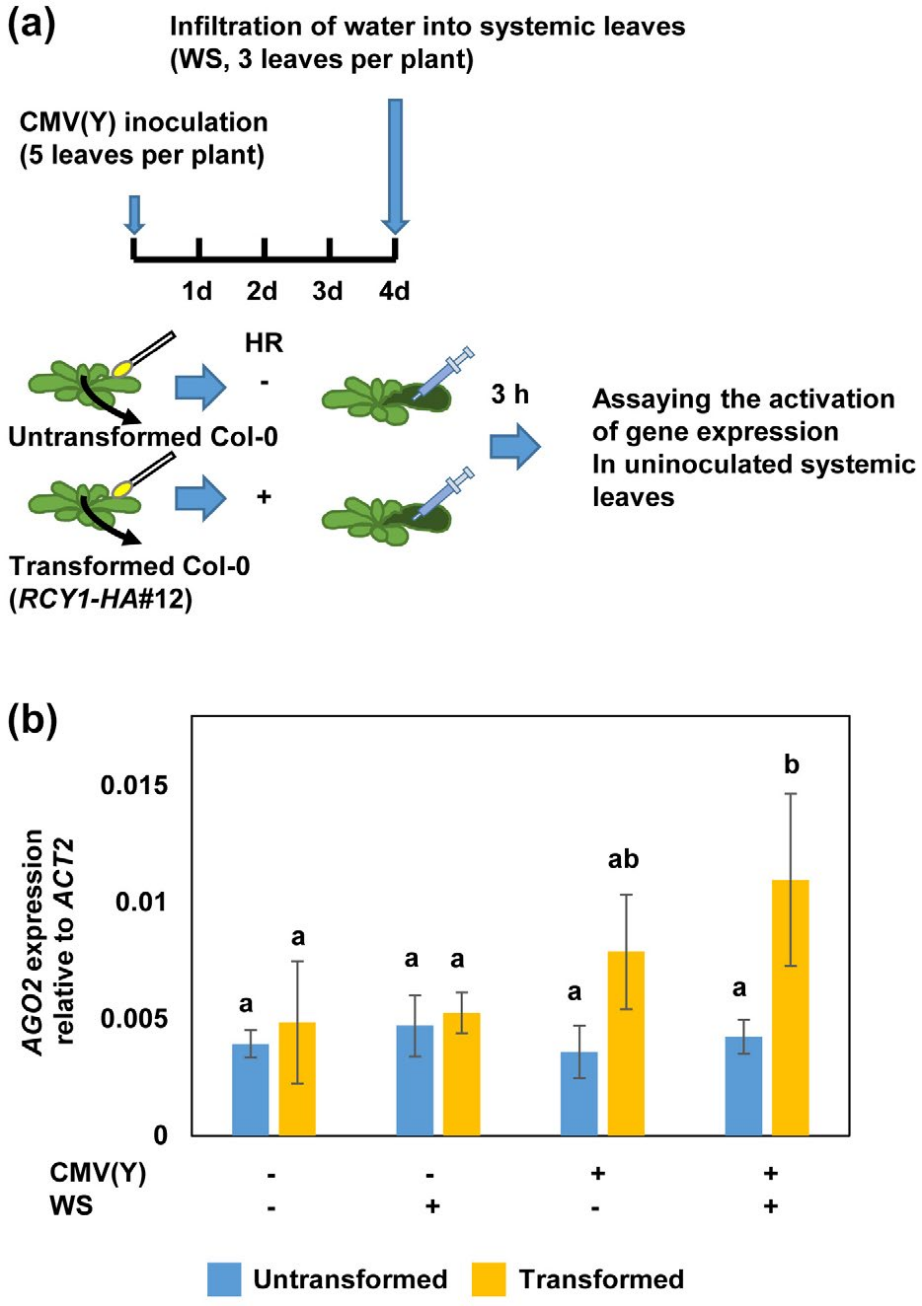
Cucumber mosaic virus (CMV[Y]) inoculation induces systemic priming for enhanced *AGO2* expression in *RCY1‐HA* expressing plants. (a) Design of the experiment. Leaves of 6‐week‐old plants of the untransformed Col‐0 and the transformed *RCY1‐HA* genotype in Col‐0 genetic background were inoculated with CMV(Y). After 4 days, uninoculated systemic leaves were left untreated (−WS) or infiltrated with water (+WS). Three hours later, the systemic leaves were harvested and analysed for the abundance of mRNA transcript of *AGO2*. (b) Different letters denote significant differences among treatments (Tukey–Kramer test, *n* = 3, *p* < .05). *ACT2*, *ACTIN2*

To investigate whether *AGO2* would also be primed for enhanced expression during pathogen‐induced SAR, we investigated the activation of *AGO2* in leaves after inoculation with CMV(Y), which is virulent for the *A. thaliana* accession Col‐0. The *R* gene *RCY1* encodes a protein with a nucleotide‐binding site and coiled‐coil and leucine‐rich repeat domain (Takahashi et al., 2002). The gene has been isolated from *A. thaliana* accession C24, which responds hypersensitively to CMV(Y) (Takahashi et al., 2002). Transformation of *HEMAGGLUTININ* (*HA*)‐tagged *RCY1* (*RCY1‐HA*) into wild‐type (Col‐0) plants was shown to provide CMV(Y) resistance (Sekine et al., 2008). To investigate whether the gene‐for‐gene resistance of *RCY1‐HA* plants to CMV(Y) is associated with priming for enhanced *AGO2* expression in systemic leaves, we tested the systemic accumulation of *AGO2* mRNA transcript upon local CMV(Y) inoculation of this genotype of plant. To do so, four leaves of the wild‐type and *RCY1‐HA* (line #12) plants were inoculated with CMV(Y). After 4 days, three uninoculated, systemic leaves were infiltrated with water. Three hours later, the abundance of mRNA transcript of *AGO2* was determined in the infiltrated leaves of both genotypes of plant. As shown in Figure 3, enhanced *AGO2* expression was seen in the infiltrated systemic leaves of the CMV(Y)‐inoculated *RCY1‐HA* plants (CMV(Y)+, WS+; yellow column) but not in the susceptible wild type (CMV(Y)+, WS+; blue column). In the systemic leaves of *RCY1‐HA* plants with local CMV(Y) infection *AGO2* was expressed also in the absence of challenge (CMV(Y)+, WS−; yellow column), but this response was lower than in the systemically challenged *RCY1‐HA* plants with local infection (CMV(Y)+, WS+; yellow column) (Figure 3). These results reveal that local activation of a CMV(Y)‐triggered hypersensitive response, in contrast to the compatible interaction, induces systemic priming for enhanced *AGO2* expression. We made similar findings when we assayed the expression of the*WRKY53* gene (Figure S4).

**FIGURE 4:**
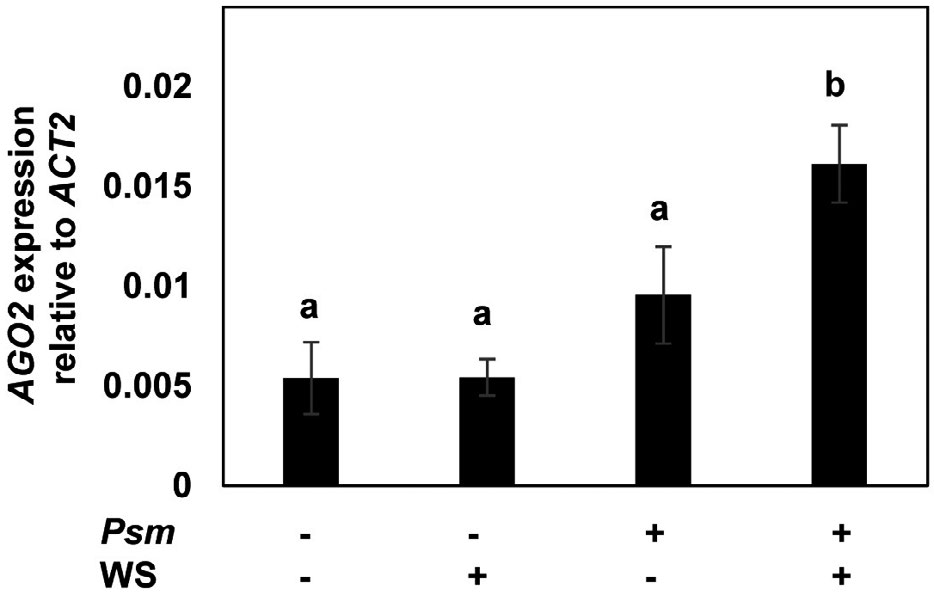
*Pseudomonas syringae* pv. *maculicola* (Psm) infection primes systemic leaves for enhanced activation of *AGO2* expression. Leaves of 6‐week‐old plants were mock‐inoculated (−Psm) or inoculated with Psm (three leaves per plant). After 3 days, uninoculated systemic leaves were left untreated (−WS) or infiltrated with water (+WS). Three hours later, the systemic leaves were harvested and analysed for the abundance of *AGO2* mRNA transcript. Different letters denote significant differences among treatments (Tukey–Kramer test, *n* = 3, *p* < .05). *ACT2*, *ACTIN2*

To further confirm the enhanced activation of *AGO2* in systemic leaves as a mechanism of biologically induced SAR, we examined the activation of *AGO2* expression in *A. thaliana* leaves upon inoculation with *P. syringae* pv. *maculicola* (Psm). As shown in Figure 4, enhanced activation of *AGO2* expression was observed in the water‐infiltrated systemic leaves of plants with local Psm infection. This result indicated that the local Psm infection triggered systemic priming for enhanced *AGO2* expression upon further stimulation.

### 2.3 Expression of *AGO2* in the *npr1* mutant

**FIGURE 5:**
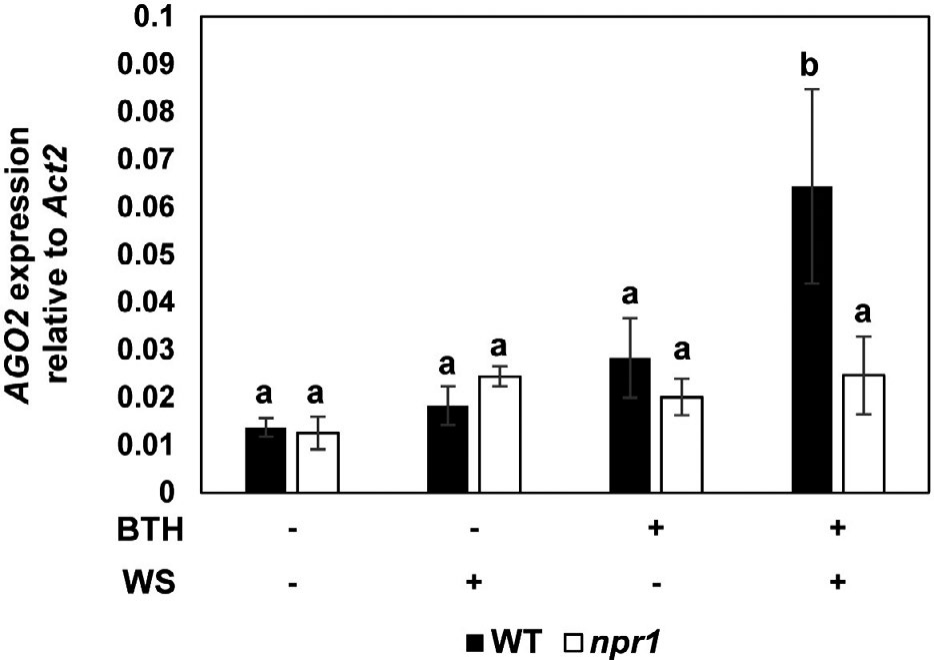
Expression of *AGO2* in response to treatments in *npr1*. Plants were treated and accumulation of *AGO2* mRNA transcript analysed as described in Figures 1 and 2. Data were normalized to the abundance of *ACTIN2* mRNA transcript. The experiments were performed at least three times. Shown is a representative result. Different letters denote significant differences among treatments (Tukey–Kramer test, *n* = 3, *p* < .05). BTH, benzo‐(1,2,3)‐thiadiazole‐7‐carbothioic acid *S*‐methyl ester. WS, infiltration of water into leaves. WT, wild type. *ACT2*, *ACTIN2*

BTH does not cause an accumulation of SA and induces disease resistance in SA‐degrading NahG plants (Friedrich et al., 1996). Therefore, the inducer was proposed to activate SAR signalling at the site of, or downstream of, SA accumulation. In addition, just like SA, BTH is unable to activate *SAR* gene expression or SAR in the *npr1* mutant, which is allelic to *nim1* (Cao et al., 1994; Delaney et al., 1995; Dong, 2004). To investigate whether priming for enhanced activation of *AGO2* (Figure 1b) would involve NPR1 and the SA signal transduction pathway, we included the *npr1* mutant in our analysis. The infiltration‐activated expression of *AGO2* was enhanced after BTH pretreatment in the wild type (Figure 1b) but it was absent from *npr1* (Figure 5). This finding indicates that the BTH‐induced priming of *A. thaliana* for enhanced *AGO2* expression requires the functional *NPR1* gene.

### 2.4 BTH treatment induces modification to histones in the *AGO2* promoter

**FIGURE 6:**
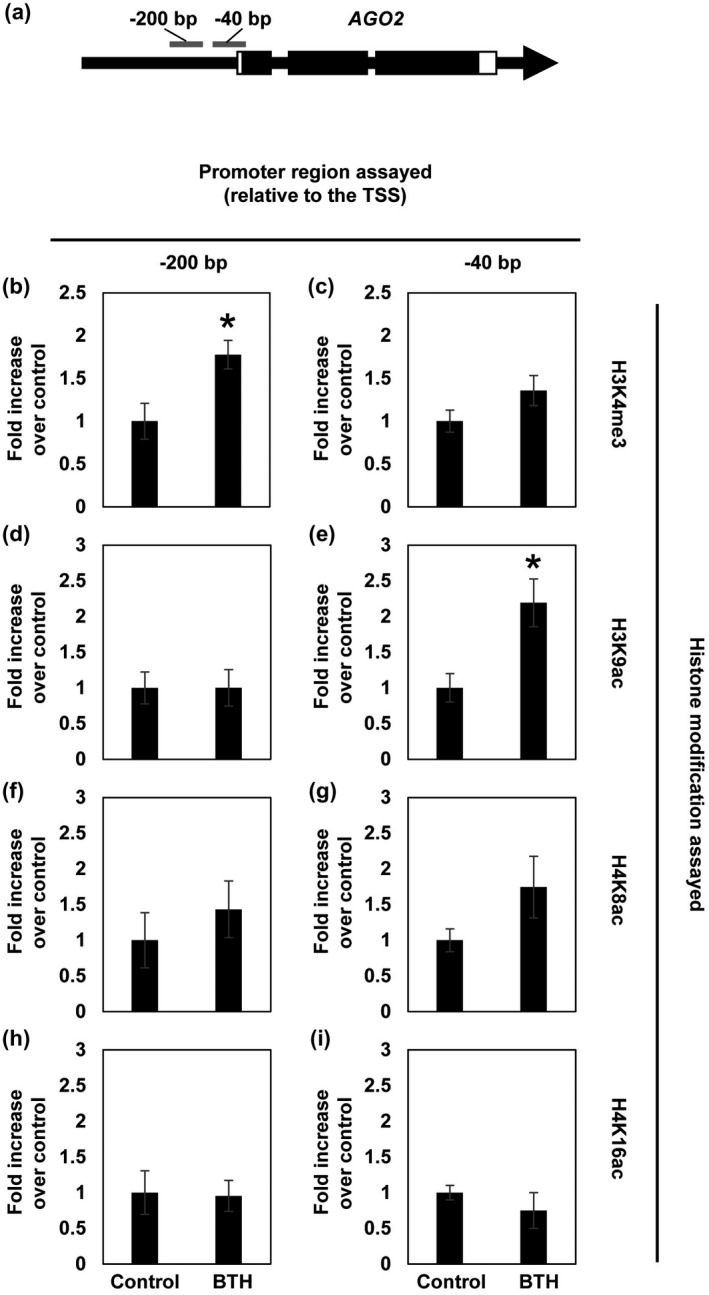
Benzo‐(1,2,3)‐thiadiazole‐7‐carbothioic acid *S*‐methyl ester (BTH)‐induced modification of histones in the *AGO2* promoter. (a) Analysed regions in the *AGO2* promoter. Arrow indicates the genomic sequence of *AGO2* and the direction of transcription. Black boxes indicate exons and white parts are untranslated regions. Leaves of 6‐week‐old plants were sprayed with a solution of wettable powder (WP) without (control) or with BTH (100 µM). After 3 days, leaves were harvested and subjected to chromatin immunoprecipitation using antibodies to the specific histone modification. Histone alterations were analysed at −200 bp (b, d, f, h) and −40 bp (c, e, g, i) relative to the transcription start site. The level of H3K4me3 (b, c) H3K9ac (d, e), H4K8ac (f, g), and H4K16Ac (h, i) was normalized to the *ACTIN2* gene. Shown is the fold increase in the modification level in BTH‐treated plants versus the appropriate WP control. Error bars indicate standard errors (*n* = 3). Asterisks denote significant differences (Student's *t* test, *n* = 3, *p* < .05). TSS, transcription start site

Some histone modifications, such as H3K4me3, H3K9ac, and H4K8ac, have been associated with the permissive state of defence genes during priming in *A. thaliana* (Jaskiewicz et al., 2011). To elucidate the molecular mechanism of *AGO2* priming, we next investigated whether priming for enhanced expression would be associated with histone modification at selected sites (−40 and −200 bp upstream of the transcription start site) in the promoter region of *AGO2*. As shown in Figure 6, the H3K4me3 mark was increased at both sites in the *AGO2* promoter. The enhancement of H3K4me3 was high at −200 bp and low at the −40 bp site (Figure 4b,c). The level of H3K9ac was especially enhanced at the −40 bp promoter site after BTH treatment, but not at −200 bp (Figure 6d,e). Histone marks H4K8ac and H4K16ac were not associated with the primed state of the *AGO2* gene, although the H4K8ac level was slightly increased at −40 and −200 bp after BTH treatment (Figure 6f–i).

The priming‐linked modification of histones in the 5′‐regulatory regions of genes is frequently associated with the formation of open chromatin in the same region (Schillheim et al., 2018; Baum et al., 2019). Therefore, we next used formaldehyde‐assisted isolation of regulatory elements (Baum et al., 2020) to examine whether BTH treatment of *A. thaliana* plants would open chromatin in the *AGO2* promoter. As shown in Figure S5, the region around the transcription start site of *AGO2*, especially at the −200 and −40 bp sites, seemed to be more open in leaves of BTH‐treated plants than in the WP controls. This finding indicates that BTH treatment induces a permissive state of transcription of *AGO2*, at least in part, by modification of histones, the associated slacking of the DNA–histone interaction, and the enhanced accessibility to chromatin of transcription‐regulatory proteins.

### 2.5 BTH‐induced resistance to CMV(Y) in wild‐type and the *ago2* mutant

**FIGURE 7:**
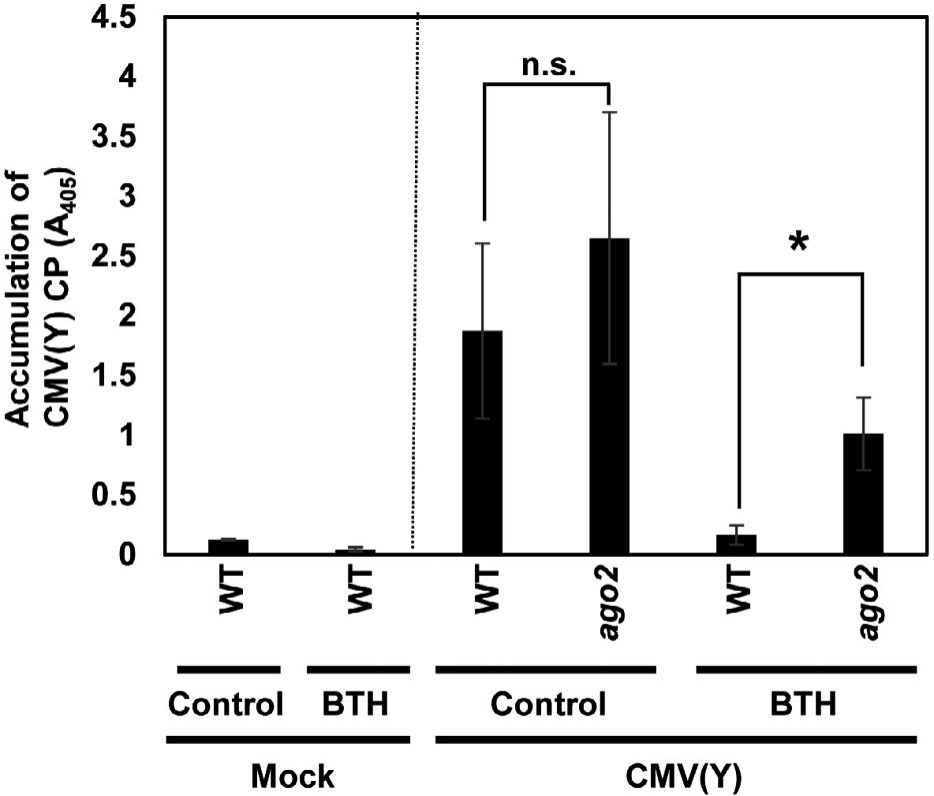
Benzo‐(1,2,3)‐thiadiazole‐7‐carbothioic acid *S*‐methyl ester (BTH) treatment reduces CMV(Y) multiplication in wild type (WT) and the *ago2* mutant. Six‐week‐old wild‐type and *ago2* plants were sprayed with wettable powder (WP) (control) or BTH (100 µM) in WP. After 3 days, leaves of the two genotypes of plants were inoculated with CMV(Y) or subjected to mock treatment (mock). After another 2 days, inoculated leaves were harvested and subjected to ELISA using an antibody against the CMV coat protein (CP). Asterisk denotes significant difference (Student's *t* test, *n* = 4, *p* < .05). n.s., no significant difference

To investigate whether the priming by BTH of *AGO2* for enhanced transcription is associated with the induction of resistance to viral infection, we examined the interaction with CMV(Y) with *A. thaliana* wild‐type plants and the *ago2* mutant (Figure 7). We quantified the amount of CMV(Y) coat protein (CP) at 2 days post‐CMV(Y)‐inoculation (dpi) of BTH‐treated and WP‐treated (control) plants by enzyme‐linked immunosorbent assay (ELISA). As shown in Figure 7, in the wild type the CMV(Y) CP level was reduced about 9‐fold after inoculation of BTH‐pretreated plants as compared to the WP controls. The decrease in viral RNA accumulation (RNA4 of CMV[Y]) in BTH‐treated plants was confirmed by quantitative reverse transcription polymerase chain reaction (RT‐qPCR) (Figure S6). In the CMV(Y)‐infected *ago2* mutant, the accumulation of CMV(Y) CP was higher than in the infected wild type, although this difference was not significant (Figure 7). The CMV(Y) CP accumulation was reduced by BTH treatment in *ago2* plants (Figure 7), although *AGO2* expression was absent (Figure S7a). The observed resistance to CMV(Y) might be caused by priming the expression of other defence genes, such as *WRKY53*, in the BTH‐treated *ago2* mutant (Figure S7b). However, when compared to the appropriate WP control in BTH‐pretreated *ago2* plants, the accumulation of CMV(Y) CP was reduced only about 3‐fold at the second day after inoculation (Figure 7). The accumulation of CMV(Y) CP in BTH‐treated *ago2* plants was significantly higher than the BTH‐treated wild type. These results indicated that the strength of the BTH‐induced resistance to CMV(Y) in *ago2* was reduced when compared to wild type.

## 3 DISCUSSION

AGO proteins are key components of the RNA silencing pathway (Carbonell and Carrington, 2015) and some of them have been shown to play a role in plant–pathogen interactions (Zhang et al., 2011; Incarbone and Dunoyer, 2013; Seo et al., 2013). For example, the CMV‐encoded 2b suppressor protein counters AGO1 cleavage activity to antagonize plant defence (Zhang et al., 2006). In addition, expression of *AGO4* is reduced by bacterial infection or after treatment with the MAMP flg22, thus implying a role of AGO4 in the antibacterial defence response of plants (Yu et al., 2013). These assumptions are consistent with the repressed accumulation of mRNA transcripts of *AGO1* (Figure 1a) and *AGO4* (Figure 1d) in BTH‐primed and unprimed plants upon water infiltration. Because AGO1 is a known suppressor of *AGO2* (Harvey et al., 2011) one might assume that suppression of the *AGO1* gene primes *AGO2* for enhanced expression. However, during priming with BTH the expression of *AGO1* is not reduced in *Arabidopsis* (Figure 1a). This finding argues against a role of *AGO1* suppression in *AGO2* priming. Yet, *AGO1* transcription is significantly inhibited after challenge of the primed plants (Figure 1a). Therefore, the post‐challenge reduction in *AGO1* expression could contribute to, or even cause, the enhanced *AGO2* expression after challenge. Because modification to histones on the *AGO2* promoter is induced already during priming (i.e., before challenge; Figure 6), we believe that histone modification is more important to *AGO2* priming than the post‐challenge inhibition of *AGO1* expression. AGO10 is known to be required for the formation of primary and axillary shoot apical meristems (Liu et al., 2009). Therefore, the reduced *AGO10* expression in BTH‐primed and unprimed plants upon challenge (Figure 1h) may reflect the plant growth‐to‐defence transition (Pajerowska‐Mukhtar et al., 2012) by turning off genes with a role in plant growth and development, including *AGO10*.

Here, we disclosed that among the *AGO* genes assayed, expression of *AGO2* and *AGO3* was faster and stronger upon challenging the leaves of BTH‐primed plants when compared to unprimed plants (Figures 1b,c and 2). This suggests that both *AGO2* and *AGO3* might contribute to the enhanced basal resistance of BTH‐primed plants to CMV(Y) (Figures 7 and S6). We assume that SAR signalling encompasses small RNAs whose action involves *AGO* genes and their proteins. *AGO4* and *AGO6* are repressed during defence priming (Figure 1d,e), whereas expression of *AGO7* is activated during priming (Figure 1f). Expression of the other tested AGO genes was unchanged during priming (Figure 1a,b,c,g,h). Together, this suggests a positive regulatory role of *AGO7* in defence priming. Upon challenge, expression of *AGO1*, *AGO4*, *AGO7*, and *AGO10* was repressed in primed plants when compared to unprimed plants (Figure 1a,d,f,h). This points to an inhibitory role of these *AGO* genes in the regulation of the augmented *Arabidopsis* defence response, possibly to avoid an exaggerated immune reaction. Correspondingly, the stronger expression of *AGO2* and *AGO3* (Figure 1b,c) points to a supporting role in the enhanced defence response of *A. thaliana*. Whether AGO2 in this process functions via the known AGO2‐miR393* pathway or whether AGO2 has different roles regarding the stimulus/inductor (SA/BTH; virus, bacteria) remains to be seen.

Consistent with the assumed positive role of AGO2 in the enhanced *A. thaliana* defence response, an *ago2* mutant is hypersusceptible to CMV (Figure 7; Harvey et al., 2011) and both *AGO2* and *AGO3* are required for the abscisic acid‐mediated resistance to bamboo mosaic virus in *A. thaliana* (Alazem et al., 2017). Because the overall abundance of *AGO2* mRNA was c.20‐times higher than transcript of *AGO3* (Figure 1b,c), we decided to investigate the role of *AGO2* in the BTH‐primed plant defence response first. The decision was strengthened by the observation that the abundance of *AGO1* mRNA transcript was markedly reduced in infiltrated leaves of BTH‐pretreated wild‐type plants (Figure 1a). Because of the predicted suppressing role of AGO1 on AGO2 function (Harvey et al., 2011) this finding supported the presumed critical role for AGO2 in SAR, although it does not exclude that AGO3 might also be important to the BTH‐induced defence priming and SAR. The different time course of *AGO2* and *AGO3* expression in primed plants after challenge (Figure 2) in fact suggests important but different roles of AGO2 and AGO3 in BTH‐induced SAR.

The enzymes RDR1 and RDR6 produce secondary viral siRNA during the induction of CMV resistance in *A. thaliana* (Deleris et al., 2006; Wang et al., 2010, 2011). In addition, *RDR1* expression can be induced by SA treatment (Yu et al., 2003). These findings link specific components of the RNA silencing machinery to SA‐induced CMV resistance. However, because SA treatment induces CMV resistance in the *dcl2/dcl3/dcl4* triple mutant, which is impaired in RNA silencing (Lewsey and Carr, 2009), SA seems to activate CMV defence in both an RNA‐silencing dependent and independent manner (Ando et al., 2019). Because BTH is a functional analogue of SA, priming *AGO2* for enhanced expression might be yet another mechanism of the SA‐induced CMV resistance in *A. thaliana*. Priming for enhanced expression was not seen for any *RDR* or *DCL* gene assayed, although BTH treatment alone seemed to induce the expression of *RDR1*, *DCL1*, and *DCL4* (Figures S2 and S3). Therefore, the enhanced activation of *AGO2* after challenge (Figure 1b) may be critical for the establishment of RNA silencing‐mediated CMV resistance in primed plants.

In *A. thaliana*, priming involves the enhanced inducibility of genes encoding transcription factors WRKY6, WRKY29, WRKY53, and PR1 associated with alterations to chromatin in the promoters of these genes (Jaskiewicz et al., 2011). Furthermore, defence priming involves an elevated level of MAMP‐recognition receptors (Tateda et al., 2014), accumulation of dormant mitogen‐activated protein kinases 3 and 6 (Beckers et al., 2009), and transcription factor HsfB1 activity (Pick et al., 2012). The work described here discloses *AGO2* as a previously unknown target of defence priming.

The enhanced inducibility of *AGO2* in primed plants was associated with modifications of chromatin in the *AGO2* promoter (Figure 6). Histone modification also accompanies the BTH‐induced priming for enhanced activation of *WRKY6*, *WRKY29*, and *WRKY53* in *A. thaliana* (Jaskiewicz et al., 2011). In addition, priming for enhanced activation of *WRKY6*, *WRKY29*, and *WRKY53*, similar to *AGO2*, was absent in the *npr1* mutant, which is defective in defence priming and SAR (Jaskiewicz et al., 2011; Figure 5). Therefore, enhanced activation of the *AGO2* gene might be regulated by one or more mechanisms that are identical to, or like, that of the *WRKY* genes. Because priming of *A. thaliana* involves the pre‐challenge modification of histones in the 5′‐leader sequence of *AGO2*, this gene, just like some *WRKY* genes, seems to be a part of the epigenetic defence memory of plants (Jaskiewicz et al., 2011; Conrath et al., 2015).

The chromatin in the *AGO2* promoter was open in the WP control and further opened slightly upon BTH treatment (Figure S5). Open chromatin formation in the *AGO2* promoter was accompanied by the induction of histone marks that have been associated with a permissive state of gene transcription (Jaskiewicz et al., 2011; Schillheim et al., 2018; Baum et al., 2019). Changes in the accessibility to regulatory DNA sites of transcription factors, which is regulated by modification to histones, eviction of nucleosomes, and the associated opening of chromatin, might thus have important roles for *AGO2* priming.

In the primed state, the expression of *AGO2* was not activated but the *AGO2* gene was ready for fast and robust activation by infiltration. Therefore, some unknown mechanism that pauses *AGO2* transcription during defence priming is likely. In *Drosophila melanogaster* and mammalian cells, transcriptional pausing often involves stalled RNA polymerase II in the promoter‐proximal region of genes (Mayer et al., 2017). In *A. thaliana*, paused RNA polymerase II is involved in the enhanced activation of drought‐response genes, being associated with high H3K4me3 and phosphorylated serine 5 in the carboxyterminal domain of RNA polymerase II (Ding et al., 2012). Therefore, the phosphorylation state of RNA polymerase II, which binds to the *AGO2* promoter, could underly the primed transcription of the *AGO2* gene.

The enhanced systemic activation of *AGO2* and *WRKY53* in plants with local CMV(Y) infection (Figures 3 and S4) suggests that the signal(s) that confer priming for enhanced *AGO2* activation seem to be translocated like the classical SAR signals (Dempsey and Klessig, 2012) or may be identical to these signals. Because tobacco mosaic virus (TMV) can induce SAR to TMV and CMV infection in certain cultivars of tobacco (Ross, 1961; Ádám et al., 2018), it would be interesting to know whether *AGO2* expression is enhanced during TMV‐induced SAR in tobacco.

To our knowledge, this is the first report demonstrating that the RNA silencing component AGO2 is associated with defence priming and SAR, and that the permissive state of *AGO2* transcription is accompanied by priming‐linked modifications of chromatin in the 5′‐regulatory region of the gene.

## 4 EXPERIMENTAL PROCEDURES

### 4.1 Plant material and chemical treatment

Seeds of *A. thaliana* wild‐type (Col‐0) plants, the mutants *npr1‐1* (Cao et al., 1994) and *ago2‐1* (SALK_003380), and the transgenic line (#12) harbouring *RCY1‐HA* that exhibits a hypersensitive response to CMV(Y) (Sekine et al., 2008), all in Col‐0 genetic background, were stratified at 4°C for 2 days. Approximately 2 weeks after sowing, seedlings were transferred to single pots and cultivated for 4 weeks in short‐day conditions (8 hr light, 23°C/16 hr dark, 18°C). Six‐week‐old plants were used in the experiments. Defence priming was induced by spray treatment with a WP formulation of BTH (Syngenta; final BTH concentration 100 µM). Spraying a WP solution without BTH (Syngenta) served as a control. Three days after spray treatment, leaves were infiltrated with distilled water as described (Beckers et al., 2009; Kohler et al., 2002).

### 4.2 Growth of pathogens and plant inoculation

CMV(Y) was propagated on *Nicotiana benthamiana* and purified as described (Takahashi and Ehara, 1993). Five leaves of 6‐week‐old *A. thaliana* plants were inoculated with CMV(Y) as described (Takahashi et al., 1994). After 4 days, uninoculated leaves were infiltrated with distilled water. The infiltrated leaves were harvested and subjected to RNA extraction 3 hr after infiltration.

Psm was propagated in King's B medium (King et al., 1954) supplemented with 100 µg/ml streptomycin at 28°C for 1 day. Bacterial cells were collected by centrifugation and resuspended in 10 mM MgCl_2_ as described (Beckers et al., 2009). Three leaves of 6‐week‐old *A. thaliana* plants were infiltrated with a Psm suspension of 5 × 10^5^ cfu/ml in 10 mM MgCl_2_ (Beckers et al., 2009). Three days postinoculation, uninoculated leaves were infiltrated with distilled water. Infiltrated leaves ware harvested at 3 hr after water infiltration and frozen until further analysis.

### 4.3 RNA extraction and RT‐qPCR analysis

Total RNA was extracted from individual leaves using the TRIzol method (Chomczynski, 1993). The relative abundance of mRNA transcript of each gene of interest was determined by RT‐qPCR using a 7300 Real‐time PCR system (Applied Biosystems). Transcript abundance was calculated and given as fold difference from *ACTIN2* (At3g18780). Then, the average and standard deviation of values of three independent leaves were calculated. Student's *t* test and the Tukey–Kramer test were performed for statistical analysis to compare two samples and three or more samples, respectively. Reverse transcription and subsequent PCR were performed using PrimeScript RT reagent Kit with gDNA Eraser (Takara Bio Inc.) and TB Green Premix Ex Taq II (Takara Bio Inc.). Each experiment was performed at least three times with similar results. The primers used in this study are listed in Table S1.

### 4.4 Quantification of CMV(Y) multiplication by ELISA

To quantify CMV(Y) multiplication, an ELISA was performed as described previously using an antibody against the CMV(Y) CP (Sekine et al., 2004). At least three independent virus‐infected leaves were collected and homogenized in GTEN buffer (10%, vol/vol, glycerol, 25 mM Tris.HCl [pH 7.5], 1 mM EDTA, 150 mM NaCl) with a 10‐fold volume of collected leaves (in terms of fresh weight). The concentration of total protein was determined in a Bradford protein assay (Bradford, 1976). Homogenates were diluted with 0.05 M Na_2_CO_3_ buffer (pH 9.6) to 0.025 mg/ml total protein and subjected to ELISA as described (Sekine et al., 2004). A rabbit antibody against CMV(Y) CP and alkaline phosphatase‐conjugated antirabbit IgG (Fc) (Promega) were used as the primary and secondary antibodies, respectively. Finally, *p*‐nitrophenyl phosphate (1 mg/ml) in AP9.5 buffer (10 mM Tris.HCl [pH 9.5], 100 mM NaCl, 5 mM MgCl_2_) was applied as a substrate of alkaline phosphatase. The absorbance of the resulting phenolate was measured at 405 nm. The amount of CP in 0.025 mg of total protein was calculated as average ± standard deviation of absorbance.

### 4.5 Chromatin immunoprecipitation

Six‐week‐old *A. thaliana* plants were treated with WP with or without BTH (100 µM) to induce defence priming. Three days later, leaves were harvested and used for chromatin immunoprecipitation (ChIP) analysis as described (Jaskiewicz et al., 2011). Antibodies against H3K4me3 (pAB‐003‐50; Diagenode), H3K9ac (07‐352; Merck), H4K8Ac (07‐328, Merck), and H4K16ac (07‐329; Merck) were used for ChIP. Precipitated DNA was quantified by RT‐qPCR and plotted as fold difference to the *ACTIN2* (At3g18780) gene. The primers used are listed in Table S1. Background signals with serum derived from rabbits that were immunized with an unrelated potato protein never exceeded 10% of positive signals.

### 4.6 Formaldehyde‐assisted isolation of regulatory elements

Formaldehyde‐assisted isolation of regulatory DNA elements from *A. thaliana* leaves was performed as described (Schillheim et al., 2018; Baum et al., 2019, 2020). Six‐week‐old *A. thaliana* plants were treated with a WP formulation in the absence or presence of BTH (100 µM). Three days later, leaves were harvested and vacuum‐infiltrated with crosslinking buffer (400 mM sucrose, 10 mM HEPES [pH 7.8], 5 mM β‐mercaptoethanol, 3% [vol/vol] formaldehyde, 0.1 mM phenylmethylsulfonyl fluoride) for DNA–protein fixation. The samples were immediately frozen in liquid nitrogen and ground with a mortar and pestle. The fine‐powdered leaf tissue (100 mg) was suspended in DNA extraction buffer (0.1 M Tris.HCl [pH 8.0], 0.1 M NaCl, 0.05 M EDTA) and sonicated in a Bioruptor bath sonicator (UCD‐250; BM Equipment Co., Ltd) at maximum output (30 s, eight times). The solution was then divided into two tubes for the formaldehyde‐assisted isolation of the regulatory elements sample and the input sample (Baum et al., 2020). DNA was extracted following the standard protocol of phenol/chloroform extraction (Dong et al., 2012). The input sample was incubated at 65°C for 6 hr to uncrosslink DNA from histone protein before DNA extraction. DNA quantification was performed by quantitative PCR using the primer sets listed in Table S1. The ratio of formaldehyde‐assisted isolation of regulatory elements DNA to input DNA was calculated, and the value normalized to the *UBIQUITIN5* gene (At3g62250).

## Supporting information


**FIGURE S1** Accumulation of *WRKY53* mRNA transcript. Leaves of *Arabidopsis thaliana* plants were sprayed with WP (control) or BTH (100 µM) in WP. After 3 days, leaves of half of the plants were infiltrated with water (water stress, +WS) or left without infiltration (−WS). Three hours later, leaves were harvested, RNA extracted and analysed for the accumulation of mRNA transcript of the *WRKY53* gene. Data were normalized to the abundance of *ACTIN2* mRNA transcript. The experiments were done at least three times. A representative result is shown. Different letters denote significant differences among treatments (Tukey–Kramer test, *n* = 3, *p* < .05). *ACT2*, *ACTIN2*

**FIGURE S2** Accumulation of mRNA transcript of *DCL* genes. Plants were treated and analysed as described in Figures 1 and S1. Data were normalized to the abundance of *ACTIN2* mRNA transcript. The experiments were done at least three times. A representative result is shown. Different letters denote significant differences among treatments (Tukey–Kramer test, *n* = 3, *p* < .05). *ACT2*, *ACTIN2*

**FIGURE S3** Accumulation of mRNA transcript of *RDR* genes. Plants have been treated and analysed as described in Figures 1 and S1. Different letters denote significant differences among treatments (Tukey–Kramer test, *n* = 3, *p* < .05). WS, infiltration of water into leaves. *ACT2*, *ACTIN2*. n.d., not detected

**FIGURE S4** Systemic activation of *WRKY53* expression after local CMV(Y) inoculation. Leaves of 6‐week‐old Col‐0 plants of the nontransgenic and *RCY1‐HA* transgenic background were inoculated with CMV(Y) (+CMV(Y)) or mock treated (−CMV(Y)). After 4 days, uninoculated, systemic leaves were left untreated (−WS) or infiltrated with water (+WS). Three hours later, systemic leaves were harvested and analysed for the abundance of mRNA transcript of *WRKY53*. Different letters denote significant differences among treatments (Tukey–Kramer test, *n* = 3, *p* < .05). *ACT2*, *ACTIN2*

**FIGURE S5** BTH treatment causes the formation of open chromatin in the *AGO2* promoter. (a) Scheme of promoter sites that we analysed for open chromatin formation. Arrow indicates the genomic sequence of *AGO2* and the direction of transcription. Black boxes indicate exons and white boxes represent untranslated regions. Red bars indicate the position of analysed sequences, and numbers indicate the position of sites relative to the transcription start site. Leaves of 6‐week‐old plants were sprayed with a solution of WP (control) or BTH (100 µM) in a solution of WP. After 3 days, treated leaves were harvested and analysed for open chromatin using formaldehyde‐assisted isolation of open chromatin. Amplicon abundance was normalized to the *UBIQUITIN* gene. Error bars indicate standard error (*n* = 3). TSS, transcription start site

**FIGURE S6** BTH suppresses multiplication of CMV(Y) RNA4*. Arabidopsis thaliana* (ecotype Col‐0) plants were sprayed with a solution of WP without (control) or with BTH (100 µM). After 3 days, leaves were inoculated with CMV(Y). Mock treatment was performed using distilled water. After another 2 days, inoculated leaves were harvested and analysed for accumulation of *RNA4* of CMV(Y), encoding the coat protein of CMV(Y). Asterisk denotes significant difference between samples in Student’s *t* test (*n* = 3, *p* < .05). *ACT2*, *ACTIN2*

**FIGURE S7** Accumulation of mRNA transcript of *AGO2* and *WRKY53* in *ago2* mutant. Six‐week‐old wild‐type and *ago2* plants have been treated as described in Figures 1 and S1. Three hours later, leaves were harvested, RNA extracted and analysed for the accumulation of mRNA transcripts of *AGO2* (a) and *WRKY53* (b) genes. Data were normalized to the abundance of *ACTIN2* mRNA transcript. Different letters denote significant differences among treatments (Tukey–Kramer test, *n* = 3, *p* < .05). *ACT2*, *ACTIN2*

**TABLE S1** Primers used in this study

## Data Availability

The data that support the findings of this study are available from the corresponding author upon reasonable request.
