# Supplementary figures and images for: Priming for enhanced ARGONAUTE2 activation accompanies induced resistance to cucumber mosaic virus in Arabidopsis thaliana
Source: Mol Plant Pathol. 2020 Oct 19;22(1):19–30. doi: 10.1111/mpp.13005 (PMC7749747; doi:10.1111/mpp.13005)

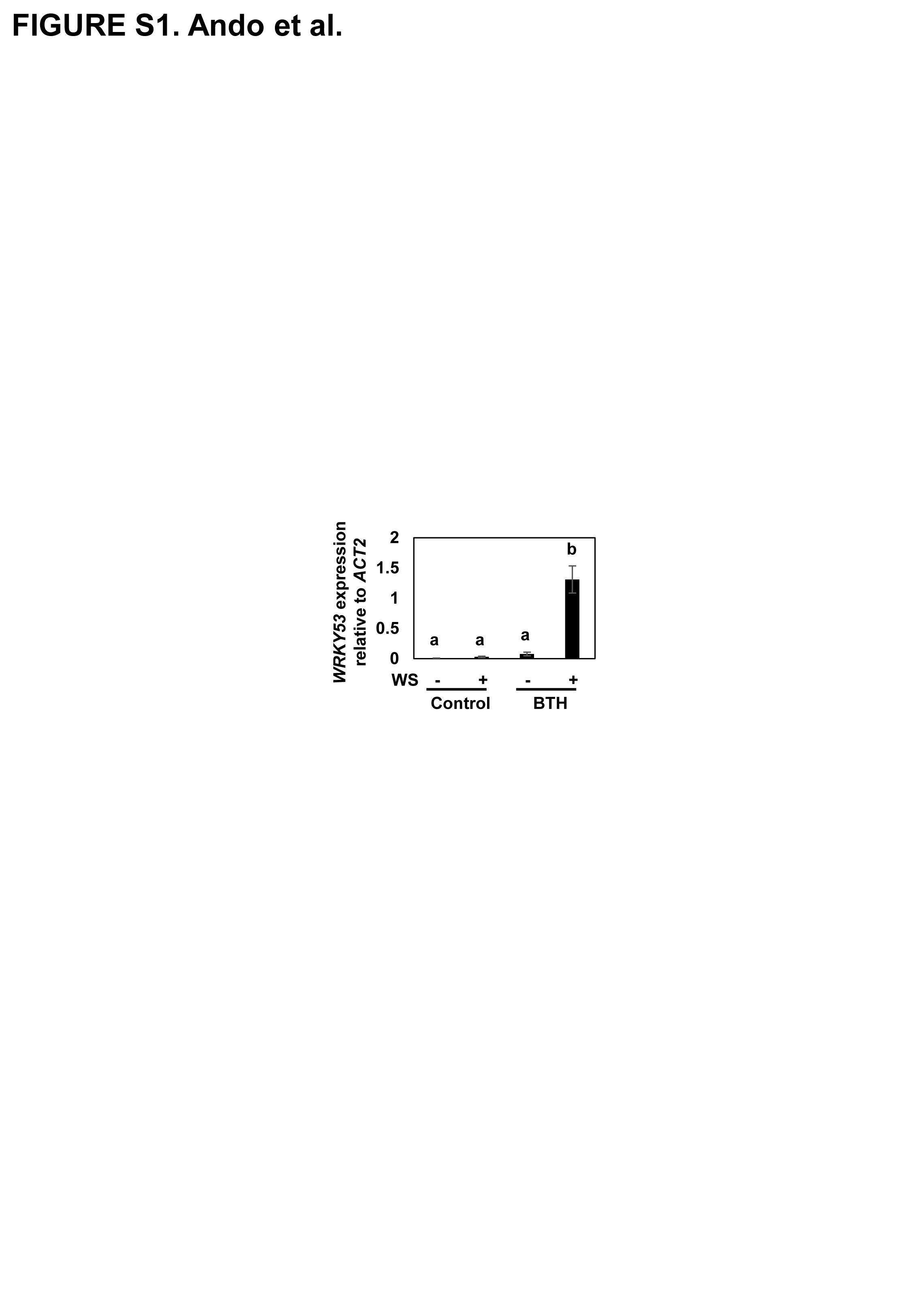

Supplement: Supplementary file 1 — FIGURE S1 Accumulation of WRKY53 mRNA transcript. Leaves of Arabidopsis thaliana plants were sprayed with WP (control) or BTH (100 µM) in WP. After 3 days, leaves of half of the plants were infiltrated with water (water stress, +WS) or left without infiltration (−WS). Three hours later, leaves were harvested, RNA extracted and analysed for the accumulation of mRNA transcript of the WRKY53 gene. Data were normalized to the abundance of ACTIN2 mRNA transcript. The experiments were done at least three times. A representative result is shown. Different letters denote significant differences among treatments (Tukey–Kramer test, n = 3, p < .05). ACT2, ACTIN2 [file MPP-22-19-s001.jpg]

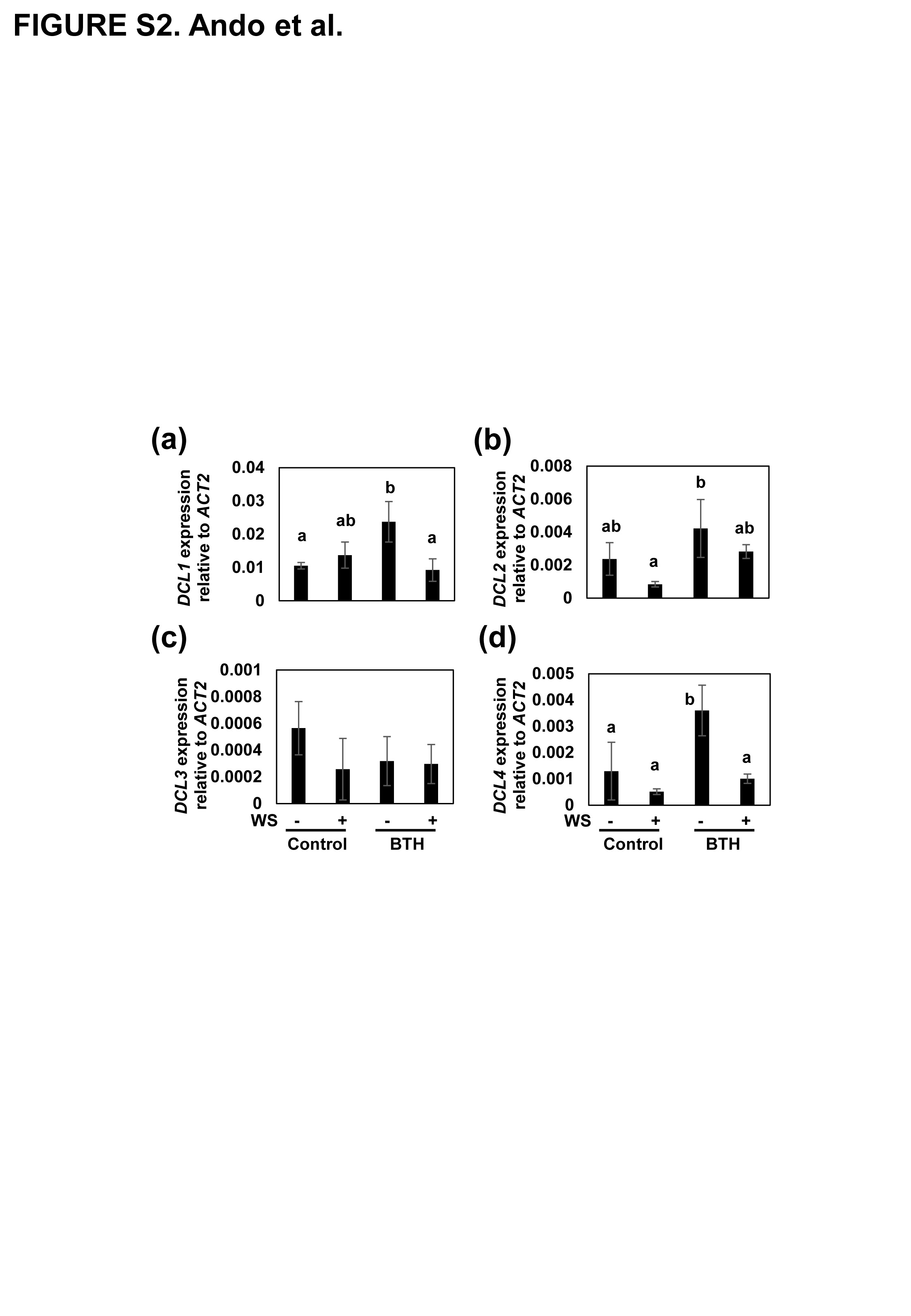

Supplement: Supplementary file 2 — FIGURE S2 Accumulation of mRNA transcript of DCL genes. Plants were treated and analysed as described in Figures 1 and S1. Data were normalized to the abundance of ACTIN2 mRNA transcript. The experiments were done at least three times. A representative result is shown. Different letters denote significant differences among treatments (Tukey–Kramer test, n = 3, p < .05). ACT2, ACTIN2 [file MPP-22-19-s002.jpg]

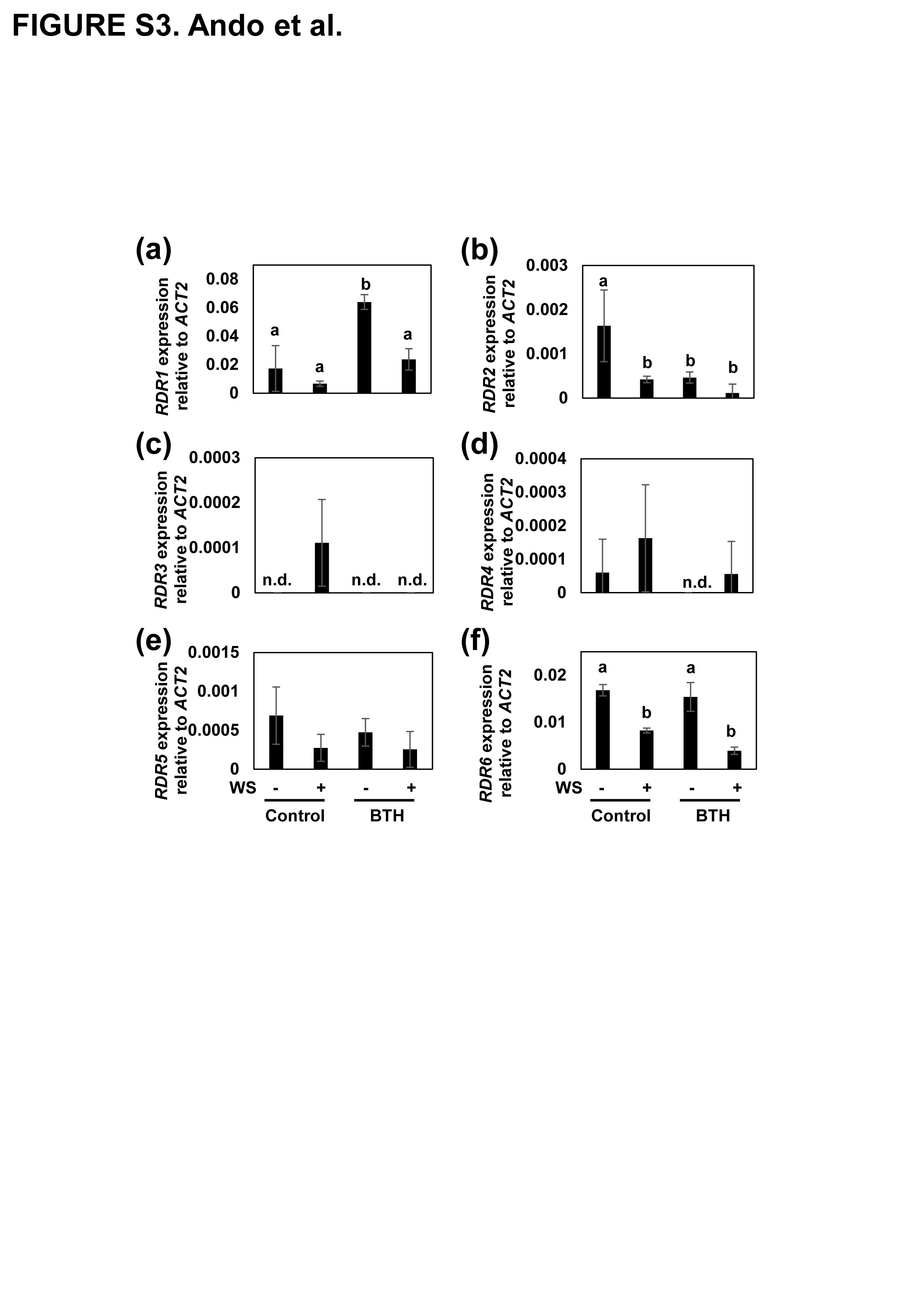

Supplement: Supplementary file 3 — FIGURE S3 Accumulation of mRNA transcript of RDR genes. Plants have been treated and analysed as described in Figures 1 and S1. Different letters denote significant differences among treatments (Tukey–Kramer test, n = 3, p < .05). WS, infiltration of water into leaves. ACT2, ACTIN2. n.d., not detected [file MPP-22-19-s003.jpg]

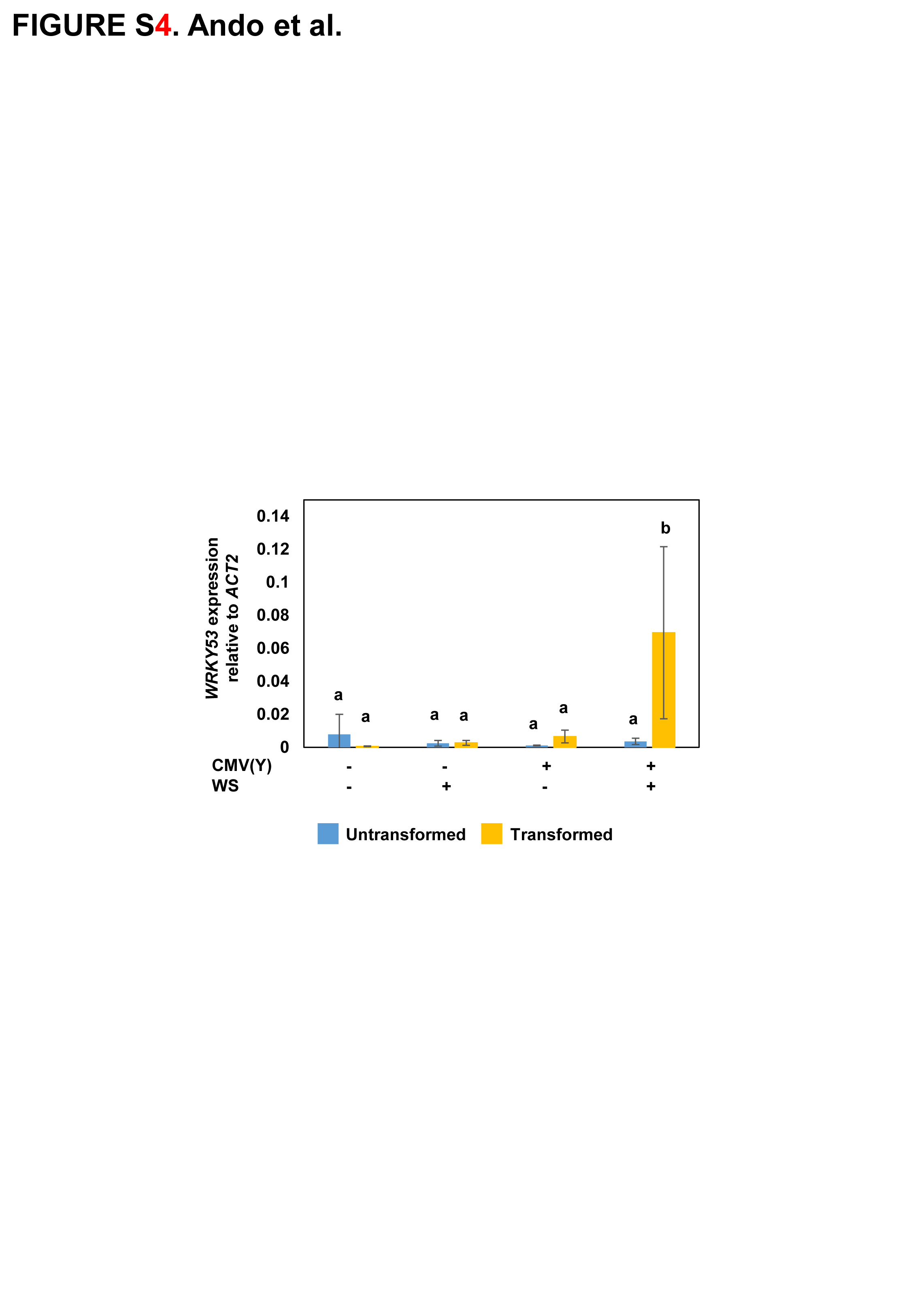

Supplement: Supplementary file 4 — FIGURE S4 Systemic activation of WRKY53 expression after local CMV(Y) inoculation. Leaves of 6‐week‐old Col‐0 plants of the nontransgenic and RCY1‐HA transgenic background were inoculated with CMV(Y) (+CMV(Y)) or mock treated (−CMV(Y)). After 4 days, uninoculated, systemic leaves were left untreated (−WS) or infiltrated with water (+WS). Three hours later, systemic leaves were harvested and analysed for the abundance of mRNA transcript of WRKY53. Different letters denote significant differences among treatments (Tukey–Kramer test, n = 3, p < .05). ACT2, ACTIN2 [file MPP-22-19-s004.jpg]

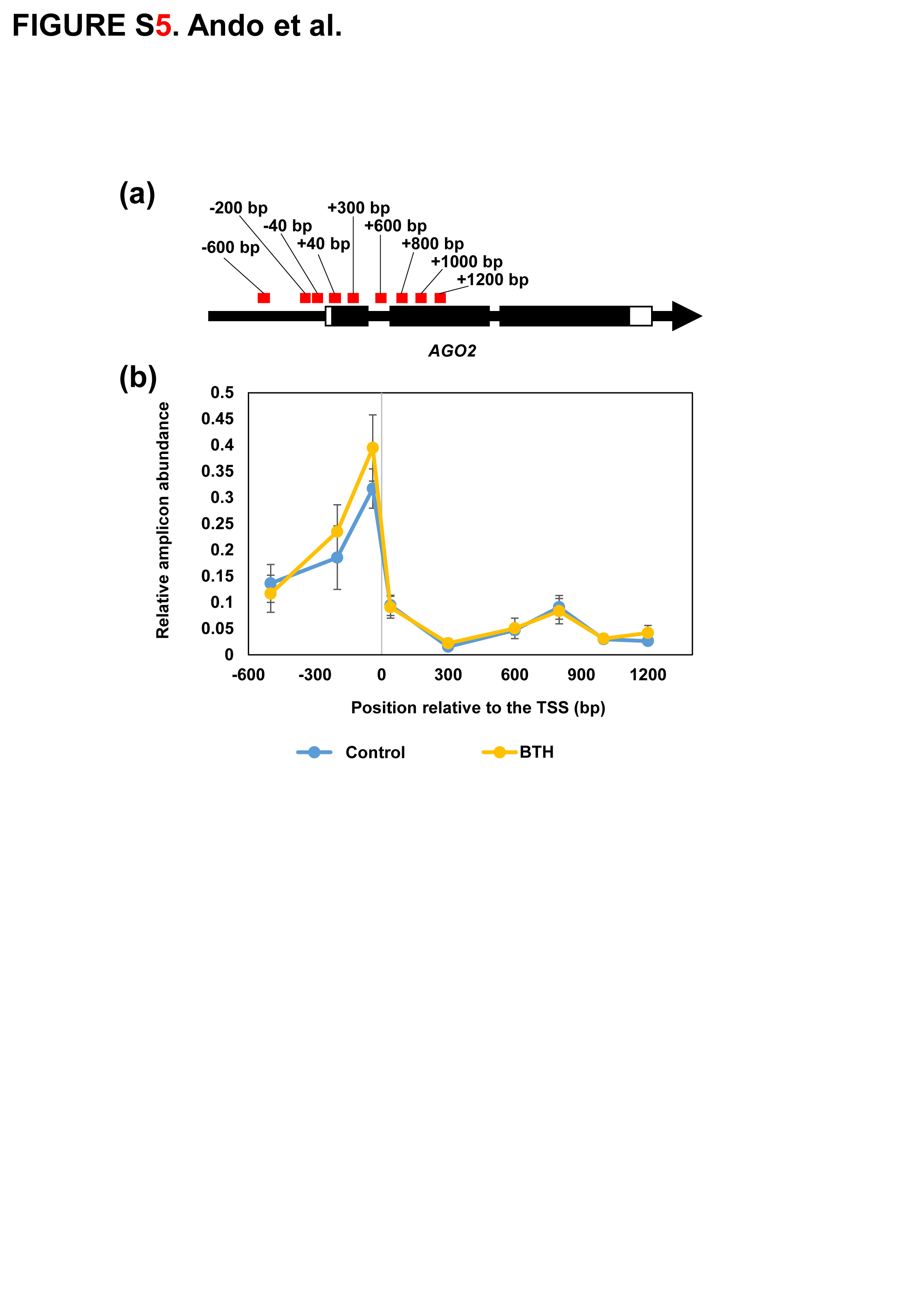

Supplement: Supplementary file 5 — FIGURE S5 BTH treatment causes the formation of open chromatin in the AGO2 promoter. (a) Scheme of promoter sites that we analysed for open chromatin formation. Arrow indicates the genomic sequence of AGO2 and the direction of transcription. Black boxes indicate exons and white boxes represent untranslated regions. Red bars indicate the position of analysed sequences, and numbers indicate the position of sites relative to the transcription start site. Leaves of 6‐week‐old plants were sprayed with a solution of WP (control) or BTH (100 µM) in a solution of WP. After 3 days, treated leaves were harvested and analysed for open chromatin using formaldehyde‐assisted isolation of open chromatin. Amplicon abundance was normalized to the UBIQUITIN gene. Error bars indicate standard error (n = 3). TSS, transcription start site [file MPP-22-19-s005.jpg]

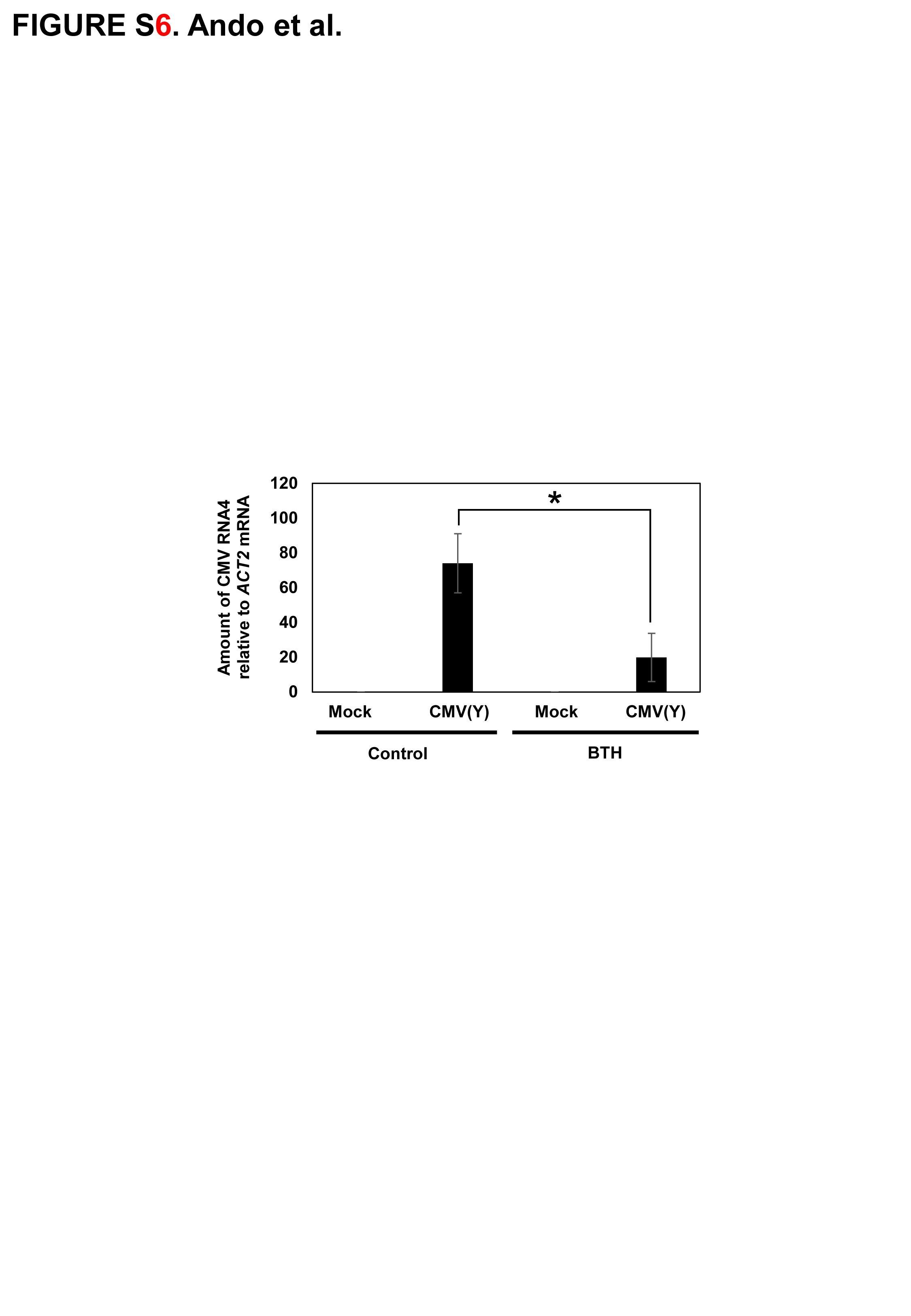

Supplement: Supplementary file 6 — FIGURE S6 BTH suppresses multiplication of CMV(Y) RNA4. Arabidopsis thaliana (ecotype Col‐0) plants were sprayed with a solution of WP without (control) or with BTH (100 µM). After 3 days, leaves were inoculated with CMV(Y). Mock treatment was performed using distilled water. After another 2 days, inoculated leaves were harvested and analysed for accumulation of RNA4 of CMV(Y), encoding the coat protein of CMV(Y). Asterisk denotes significant difference between samples in Student’s t test (n = 3, p < .05). ACT2, ACTIN2 [file MPP-22-19-s006.jpg]

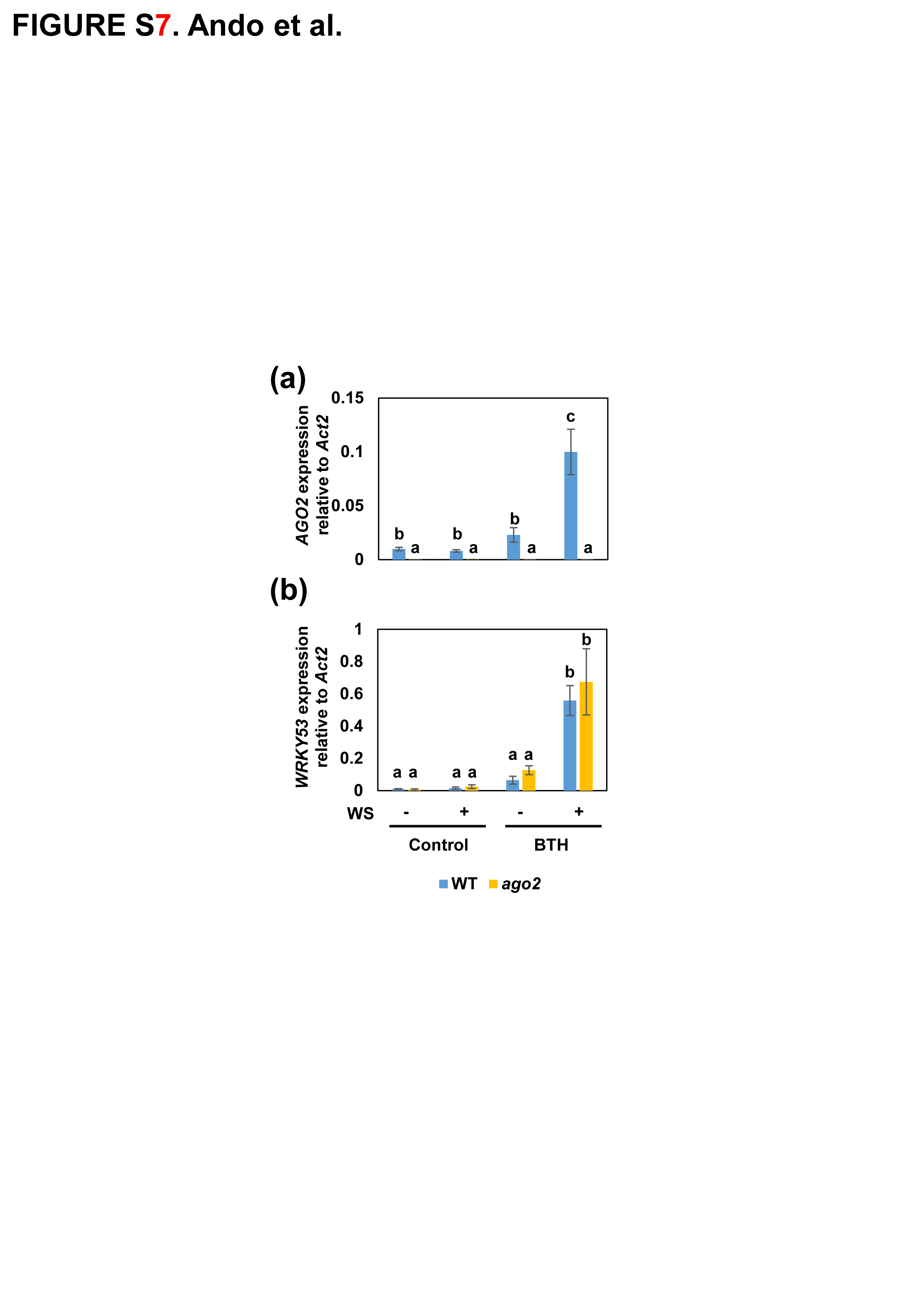

Supplement: Supplementary file 7 — FIGURE S7 Accumulation of mRNA transcript of AGO2 and WRKY53 in ago2 mutant. Six‐week‐old wild‐type and ago2 plants have been treated as described in Figures 1 and S1. Three hours later, leaves were harvested, RNA extracted and analysed for the accumulation of mRNA transcripts of AGO2 (a) and WRKY53 (b) genes. Data were normalized to the abundance of ACTIN2 mRNA transcript. Different letters denote significant differences among treatments (Tukey–Kramer test, n = 3, p < .05). ACT2, ACTIN2 [file MPP-22-19-s007.jpg]
