# Supplementary material for: Priming for enhanced ARGONAUTE2 activation accompanies induced resistance to cucumber mosaic virus in Arabidopsis thaliana
Source: Mol Plant Pathol. 2020 Oct 19;22(1):19–30. doi: 10.1111/mpp.13005 (PMC7749747; doi:10.1111/mpp.13005)
Supplement: Supplementary file 8 — TABLE S1 Primers used in this study [file MPP-22-19-s008.docx]

| Table S1. Primers used in this study | | | |  |  |
| --- | --- | --- | --- | --- | --- |
| Gene | Accession | Forward primer (5'-3') | Reverse primer (5'-3') | Application | |
| ACT2 | AT3G18780 | GGTAACATTGTGCTCAGTGGTGG | GGTGCAACGACCTTAATCTTCAT | qRT-PCR, ChIP | |
| AGO1 | AT1G48410 | AGGTGGAATGGCTGGTAGAAG | GAGGGTGAATCAACTCAGCAG | qRT-PCR | |
| AGO2 | AT1G31280 | GGACAAAGCGATTTTCAAGC | TCAGCTTGAGACCCAAAACAC | qRT-PCR | |
| AGO3 | AT1G31290 | TAGGGATGGTGGTTGGGTAA | GCCTTGACTCACTTGAGCTTG | qRT-PCR | |
| AGO4 | AT2G27040 | ATGGTGGTATCACAGCTCCAG | CCAAGTAGGGCTGCTCATTTA | qRT-PCR | |
| AGO5 | AT2G27880 | GCAGCTCCCAGCAATAAAAG | ACAGCCAAACCCAAAAGAGA | qRT-PCR | |
| AGO6 | AT2G32940 | ACGATACGCTCATCTTGCAG | TTCGACATTCTCGTGCAGAC | qRT-PCR | |
| AGO7 | AT1G69440 | CTCACCTTGCTGCGTACAGA | GAATCGTCTTTGGAGGACCA | qRT-PCR | |
| AGO8 | AT5G21030 | TCTCCATACCATGTCGTCTTTG | GGAACTTCGCCGAGTATTGA | qRT-PCR | |
| AGO9 | AT5G21150 | CTAGCAGAAATCACGCTGGA | GGGGATCTTTGCAGCATAAC | qRT-PCR | |
| AGO10 | AT5G43810 | TCATCTTGCAGCATTTCGAG | TCCGACAGTTGTTGTTTTCG | qRT-PCR | |
| DCL1 | AT1G01040 | GGTGGAGGCTTACCAAACAA | TTTGCAACCCATTCTCCTTC | qRT-PCR | |
| DCL2 | AT3G03300 | GAAGTGGCAGTTCGAGTTGAG | TTATCAGAGGCCTTTGCTGTG | qRT-PCR | |
| DCL3 | AT3G43920 | GCCTCGTGGAACTCTACATGA | CTGAAGCTAGTCCGCTTCTCA | qRT-PCR | |
| DCL4 | AT5G20320 | TAGATTTTGGAGCGGAGAGC | GCCTATCATGCTATGGGTTCA | qRT-PCR | |
| RDR1 | AT1G14790 | GGAATTGGGATGAGGAAACTG | AGCACCAATCACCTGAATCAC | qRT-PCR | |
| RDR2 | AT4G11130 | GGATGGTTCGAGAAAAGCTG | ACGATCCATGGGAAACTCAA | qRT-PCR | |
| RDR3 | AT2G19910 | AATGGCTGCGATGAAGTCTC | AAGTTTTCGCCTTGTCTCCA | qRT-PCR | |
| RDR4 | AT2G19920 | CGTGGTCAGAAATTGCTCTTC | TCGAAGAAAGTCTCCGTCTTG | qRT-PCR | |
| RDR5 | AT2G19930 | GGGTTTTGAGTTGGTCAGGA | TGTCTTCCAGAGGGGCTAAG | qRT-PCR | |
| RDR6 | AT3G49500 | GGGGAATCCACTACTGCAAA | TAAGACCCAGGAGGAGTCCA | qRT-PCR | |
| WRKY53 | AT4G23810 | CTCCATCGGCAAACTCTTCAC | CCGAGCGTACAACTTATTCCG | qRT-PCR | |
| CMV CP | M57602 | GGGTGACAGTCCGTAAAGTTTC | TGATAAACCAGTACCGGTGAGG | qRT-PCR | |
| AGO2 | AT1G31280 | CCACACCCATTCAAGGTCTTA | GGGATGTTGAATCGTCTTTCA | ChIP (-200), FAIRE (-200) | |
| AGO2 | AT1G31280 | GGGACAAGTTCTCTCTGTTTTTC | TGAACTAAACCAAATCGGAGGT | ChIP (-40), FAIRE (-40) | |
| AGO3 | AT1G31290 | AATGCCACGTCTTACAGTTGC | AATCTTCCACGAAACCACCA | ChIP (-200) | |
| AGO4 | AT2G27040 | GCCGTGACGTAGAGGAACA | TAGCTCCGACCTGAGAGGAA | ChIP (-30) | |
| AGO2 | AT1G31280 | GATGTGTTGCAAGGAATGGA | CATCTGGCTCAGTTTCACGA | FAIRE (+1200) | |
| AGO2 | AT1G31280 | TTCACTGACAATCCCGATGA | CCACCTTGTATGACCCCGTA | FAIRE (+1000) | |
| AGO2 | AT1G31280 | GTGCAAGTTGCGACTTCTGA | TAGATTAACACGCCGCACAG | FAIRE (+800) | |
| AGO2 | AT1G31280 | GGGAAATGAAATTAGGGCAAA | CAACTTTGAGCTAAACAATAATCACA | FAIRE (+600) | |
| AGO2 | AT1G31280 | CAGGATTTTCGAAGCCAGAG | CCTCCTACTGAACCGGCATA | FAIRE (+300) | |
| AGO2 | AT1G31280 | GCAGCAAAATTCTGAACACG | CACCACGACCTCCTCGATAA | FAIRE (+40) | |
| AGO2 | AT1G31280 | TCTTGGTTTTTGTTTTTGGTGTAG | TCATAAAACCATCCATTACATTCTT | FAIRE (-600) | |
| UBQ5 | AT3G62250 | GTGAGCTTAATGGCCCAATAGACT | CGTTACTACCCTTATTTTATTTGGGCT | FAIRE | |
